# Patient expectations of outcome from systemic treatment for metastatic breast cancer

**DOI:** 10.4314/gmj.v60i1.2

**Published:** 2026-03

**Authors:** Anita E Agbeko, Adelaide Bekai, Jonathan Boakye-Yiadom, Serwaa Asare-Bediako, Jonathan Bayuo, Francis Aitpilla, Kwabena Agbedinu, Ishmael Kyei, Joshua Arthur

**Affiliations:** 1 Department of Surgery, School of Medical Sciences, Kwame Nkrumah University of Science and Technology, Kumasi, Ghana; 2 Directorate of Emergency Medicine, Komfo Anokye Teaching Hospital, Kumasi, Ghana; 3 School of Public Health, Kwame Nkrumah University of Science and Technology, Kumasi, Ghana; 4 Directorate of Surgery, Komfo Anokye Teaching Hospital, Kumasi, Ghana; 5 School of Nursing and Midwifery, University of Allied Health Sciences, Ho, Ghana; 6 Disease Surveillance Department, Ghana Health Service, Accra, Ghana

**Keywords:** Expectation, Outcome, Metastatic, Breast cancer, Treatment

## Abstract

**Objectives:**

The primary goal of treatment of metastatic breast cancer (MBC) is to improve patients' quality of life (QoL). This study aimed to explore the expectations of patients with metastatic breast cancer receiving systemic therapy.

**Design:**

A longitudinal qualitative approach was used in this study.

**Setting:**

The study was conducted in the breast clinic and the oncology department of a tertiary facility in Ghana.

**Participants:**

Fifty-six (56) women diagnosed with MBC aged between 18 and 75 years participated in the study

**Intervention:**

In-depth interviews were conducted to explore participants' expectations regarding the systemic treatment they received before its initiation and 3 months after its initiation. The data was analysed thematically.

**Results:**

At the start of treatment, participants expected to be healed or cured, to avoid death, to experience improvement in their symptoms, and to be able to perform their usual functions/roles. At three months follow-up, 50% of the women had died. Of the 28 who were alive, some had experienced improvement in their symptoms, some had no change, and others had deteriorated. The cost of treatment was a concern.

**Conclusion:**

Women with MBC have unfeasible expectations of a cure from the treatment they receive. After starting systemic treatment for their disease, symptoms improved for some participants, while others deteriorated. Mortality among women with MBC is high.

**Funding:**

None declared

## Introduction

Cancer is a leading cause of death in both developed and underdeveloped countries, and this burden is expected to increase all over the world as a result of growth and ageing populations, especially in less developed countries.^1^ Breast cancer is the most common cancer in women, and at least 50% of breast cancer occurrences and 62% of deaths from it occur in less-developed countries.^1^ In spite of developments made in breast cancer screening for early diagnosis and treatment, 20-85% of women, based on their tumour biology, stage of disease and treatment used, will go on to develop distant metastasis^2^ as a result of subclinical micrometastasis in spite of appropriate and adequate primary treatment. Another 6-10% of women will have metastatic disease at primary diagnosis^3^ to the bones, liver, lung and brain in isolation or in combination.^4^,^5^ The current aggressive treatment modalities for breast cancer have impacted treatment outcomes for all stages of the disease.^2^,^3^,^6^ But metastatic breast cancer (MBC) is incurable, and although some women live for several years with a good quality of life, the average survival is 1-2 years.^7^ The 5-year survival is reported to be nearly 27% in developed countries, with up to 9% living for 10 years.^8^ In Kenya, 3 and 5-year survival for MBC has been reported as 31.3% and 10.7%, respectively.

The benefit of chemotherapy in the setting of advanced cancer has historically been defined by overall response rate, time to tumour progression, and overall survival.^9^ Patient performance status has often been used as an indicator of a patient's ability to tolerate chemotherapy as well as response to treatment, and has been used to measure the potential clinical value of chemotherapy.^10^

An Eastern Cooperative Oncology Group (ECOG) status of 2 or more suggests a survival prognosis of less than 6 months and indicates that treatment has probably not improved survival.^11^ However, medical decision making on when to stop chemotherapy remains a very subjective, intimate and complex process, and physicians have expressed concerns about the benefits of chemotherapy for cancer patients who are nearing death.^9^,^12^–^14^ In recent times, patients seek greater dialogue with their physicians to discuss and understand treatment options, side effects, complementary therapies and pain control, access to adequate psychosocial support and the reassurance that they are receiving the best available therapy.^8^, ^15^ Together with patients diagnosed with metastatic cancer, physicians must often choose between the possible benefits of chemotherapy, such as symptom relief and prolonged survival and its possible detrimental effects on quality of life. Patient perceptions of the benefits or not of treatment affect their decision making. This study explored the expectations of women with MBC undergoing treatment at KATH.

## Methods

### Study design and setting

We conducted a longitudinal qualitative study among women with MBC receiving treatment at Komfo Anokye Teaching Hospital (KATH). The longitudinal design afforded the opportunity to assess change over time, that is, whether the treatment had resulted in the anticipated expectations expressed at the start of treatment.^16^ A cross-sectional design, with a single data collection point, would not demonstrate this.^17^

KATH is a 1,400-bed capacity teaching hospital located in Kumasi, the capital of the Ashanti Region of Ghana. It serves as a major cancer referral centre for almost 50% of the Ghanaian population, with up to 35% of cancer cases seen in KATH being breast cancer. About 85% of breast cancer patients present with stage III/IV disease.^18^ Breast cancer management in KATH is a multidisciplinary effort involving pathologists, general surgeons, radiologists, oncologists, social workers, and nurses.

### Participants, recruitment and sampling

Women aged 18-75 years with MBC who had been enrolled in care at KATH were eligible for inclusion in this study. All patients with Stage I, II or III breast cancer were excluded. The presence of metastasis among breast cancer patients was determined by chest computed tomography (CT) scan (lung), abdominal ultrasound (liver) and x-rays (bones). Brain CT scans were performed in patients with a clinical indication. Occasionally, the tumour board accepted for patients with respiratory symptoms who were unable to afford a chest CT scan to take a chest x-ray to ascertain lung metastasis. Women who had already commenced their chemotherapy were excluded.

To identify potential participants, a member of the research team attended each weekly tumour board meeting as an observer to compile a list of women with MBC who were presented at the meeting. Potential participants were approached face-to-face during their first oncology clinic appointment to explain the study's purpose and objectives and invite them to consider participating. A member of the research team followed up afterwards, and if they agreed to participate, the informed consent process was completed.

### Data Collection

Each participant was interviewed twice, that is, before and after commencement of treatment. The initial interview was scheduled at the participant's convenience, before any treatment was started. At the initial interview, participants were asked, “What are your expectations from the treatment you are about to begin?” Another appointment was scheduled 3 months after the initial interview, at which the question asked was, “Have your expectations from the treatment you received been met?” The second interview was scheduled as such because, all things being equal, participants would have received at least 3 cycles of systemic cancer treatment by 3 months. Such a timeline is considered suitable to assess the effects of the treatment received.^19^ Participants who received supportive treatment only had their second interview at the same period to match those receiving systemic cancer treatment.

### Data analysis

Interviews were conducted in the Twi dialect, with translation to English during transcription. Following translation and transcription, 2 research team members (AEA and AB) read and re-read the transcripts to obtain an overall sense of the participants' expectations before and after commencing treatment. The data were coded manually in Microsoft Word and analysed using an inductive thematic approach by two research team members (AEA and AB). The thematic analytical approach sought to discover, understand, and report patterns in the data through the following steps: becoming familiar with the data, generating initial codes, searching for themes, reviewing themes, defining themes, and writing up.^20^ The first 10 transcripts were individually coded by AEA and AB. Both then compared their individually assigned codes to ensure consistency and reliability in the coding process. A coding framework was developed and applied to the remaining transcripts. New responses that fell outside the framework but were identified as important to the research question were also captured to ensure no insights were overlooked.^20^,^21^ Reiteratively, discrepancies were discussed and resolved through consensus to ensure the codes accurately reflected the participants' responses. The codes were checked for patterns, consistency and variability. Recurring concepts, indigenous terms and metaphors, and language connectors describing relationships were used as a guide to identify themes.^22^ Similar codes were aggregated to develop subthemes and themes.

### Ethical considerations

Ethical approval for the study was obtained from the Kwame Nkrumah University of Science and Technology School of Medical Sciences/Komfo Anokye Teaching Hospital Committee on Human Research, Publication and Ethics (CHRPE) in Ghana, with reference number CHRPE/AP/557/18, issued on 25^th^ September 2018.

Having MBC made the study participants potentially vulnerable, which posed ethical concerns. Potential participants were met at their first oncology clinic appointment, and the study purpose and procedures were explained in detail using the Participant Information Leaflet as a guide. The initial meeting was held in the presence of any accompanying caregiver, but without health staff, to ensure that participants did not feel that their participation or nonparticipation would influence their treatment in any way. The possibility of interviews provoking anxiety or distress was explicitly explained, and if such should occur, the interview would be suspended and only completed later if the participant indicated willingness to continue. No such situation occurred with any of the participants interviewed. Interviews were conducted in a quiet room in the oncology department, using a dedicated recorder kept in a locked drawer in a locked office to ensure confidentiality. Interview transcripts were labelled with codes and not patient names to further ensure confidentiality. Willingness to participate was indicated by a signature or thumbprint on a consent form.

## Results

### Participant characteristics

Fifty-six women with MBC were recruited from July 2019 to November 2021. The age of the women ranged from 23 to 88 years, with a mean of 51.7 years (SD 13.8). Thirteen (23.2%) were less than 40 years, 12 (21.4%) were aged 40 - 49 years, and 31 (55.4%) were aged 50 years and above. Twenty-five (44.6%) of the women were traders, and 11 (19.6%) were unemployed. Seven (12.5%), 6 (10.7%), 4 (7.1%), 2 (3.6%), and 1 (1.8%) were pensioners, artisans, farmers, public servants and a student, respectively. Twenty-six (46.4%) were married and 13 (23.2%) were widows. Clinicopathological characteristics of the participants are presented in Table 1.

**Table 1:** Clinicopathological characteristics of study participants

Tumor characteristic	Frequency (%)	Immunohistochemistry status	Frequency (%) N = 29
**Stage (T)**	**N = 56**	Luminal A (ER+ and/or PR+) HER2-)	7 (24.1%)
**T2**	1 (1.79%)	Basal-like (ER- PR-HER2-)	12 (41.4%)
**T3**	4 (7.1%)	HER 2 positive (ER- PR-HER2+)	10 (34.5%)
**T4a**	10 (17.9%)		
**T4b**	23 (41.1%)	**Metastatic site**	**N = 56**
**T4c**	18 (32.1%)	Visceral only	46 (82.1%)
		Bone only	3 (5.4%)
		Bone + visceral	7 (12.5%)

**Stage (N)**	**N = 56**	**Number of Metastatic sites**	**N = 56**
**N1**	13 (23.2%)	Single metastatic site	29 (51.8%)
**N2**	21 (37.5%)	Two metastatic sites	20 (35.7%)
**N3**	22 (39.3%)	Three metastatic sites	7 (12.5%)

### Treatment received

Regarding treatment received, 27 (48.2%) of the participants received some systemic cancer therapy during the study period. Of these, 14 received three cycles of treatment. Five (5) had 3 cycles of Adriamycin and Cyclophosphamide; 4 had 1 cycle of Adriamycin and Cyclophosphamide plus 2 cycles of Paclitaxel; 1 had 3 cycles of Carboplatin; 1 had 3 cycles of Paclitaxel; and 1 had 3 cycles of tamoxifen. Two (2) had 2 cycles of systemic therapy (1 = Adriamycin and Cyclophosphamide; 1 = Herceptin), and 11 (39.3%) received only 1 cycle (Adriamycin and Cyclophosphamide). Twenty-nine (51.8%) of the women received no systemic cancer treatment but supportive treatment only (pain and other symptom control) based on their clinician's assessment and treatment decision.

### Patients' expectations prior to commencing treatment

Three themes emerged from the responses given to the question “What are your expectations from the treatment you are about to begin?” These were 1) Curative expectations; 2) Symptom improvement; and 3) Role restoration.

#### Curative expectations

The start of the cancer treatment process was marked with hope and expectations of attaining a cure for the disease. Most participants said they expected/looked forward to healing from the treatment they were about to undergo. They held on to the expectation that the cancer would leave their bodies completely:

*“I hope to receive healing, and I won't see the illness anymore in my body” – 0001; 0039*

*“I want to be healed and free” - 0003; 0005*

*“Be healed completely” – 0037; 0053*

*“I am looking forward to be healed” – 0017; 0018 0020; 0021; 0023; 0024*

A few of the women expected that the treatment would stop the cancer from killing them.

*“I am expecting that the cancer won't kill me” – 0002*

*“I want to live longer and take care of my children” – 0010*

*“I want cure and not die, the disease will go away completely, and I will not die” – 0016*

#### Symptom improvement

Some of the women expected symptom improvement upon initiating treatment. They expected their pain to reduce or be gone, a reduction in the swelling in their arm and in their breast, to breathe better and to regain their energy.

*“If my swollen hand is reduced, I will be happy” – 0004*

*“My hardened breast will soften and my swollen hand reduce” – 0005*

*“I don't experience the pain and tiredness any longer” – 0008*

*“I want my breast to be cut off so the pain will reduce, so the pain goes away” – 0011*

*“I hope the swollen breast come back to normal size” – 0013*

*“I want that the treatment heals me from the waist pains” – 0021*

*“To breathe well again and regain all my energy back, I want that” – 0040*

### Role restoration

The hope of returning to their usual social roles and functions was another expectation of the treatment. These included attending social gatherings, cooking, looking after their children, and returning to their occupation.

*“…I want to be able to attend any social gathering” – 0013*

*“… if I can just regain my strength, even get strength to cook for my family, that would be good” – 0014*

*“.. taking the treatment so that I get the strength to look after my kids” – 0017; 0018; 0026; 0045; 0054*

*“..hmm, I need more energy to continue my business and enjoy life” – 0043; 0056*

*“..so I can go back and work on my farm, that will be very good for me” – 0044*

### Participants' expectations three months after commencement of treatment

At the follow-up interview, 28 of the initial 56 participants were alive and were interviewed. Nine of them received supportive treatment for symptom management only, with no systemic cancer treatment.

The themes emerging were related to 1) improved health, 2) deteriorated health, and 3) financial burden.

### Improved health

Some of the women admitted their health status had improved, with decreased severity of pain, and improvement in general wellbeing, activity and how their breasts looked.

*“The treatment has helped me regain some strength compared to when I first came to the hospital” – 0001*

*“I have seen so much improvement in my health, I thank God, and I am expecting more” – 0002*

*“I feel my condition has improved because I have stopped experiencing dizziness and all bodily pains” – 004*

*“My breast has now softened and the pain has decreased” – 0012*

*“I am much better now, the pain has decreased and I am hoping for more improvement as I continue with the treatment” – 0017; 0019*

A few of them expressed a sense of satisfaction even though their symptoms had not completely resolved

*“I am better now. I was very scared in the beginning but I am happy now” – 0034*

*“I still feel pain but there is a lot of improvement” – 0039*

### Deteriorated health

Despite improved health status reported by some participants, others felt their health had not changed or had deteriorated, such as increased pain and a feeling of weakness despite treatment.

*“my condition has worsened, the pain keeps increasing day by day” – 0009*

*“I have not seen any change, I think my condition is worsening” – 0011*

*“I am getting weaker by the day” – 0022*

*“I feel I am getting worse, at first I wasn't feeling this weak” -0027*

*“I have not seen much change but I am hoping to get better” – 0045*

One woman said her condition had worsened because the breast lump was not removed.

*“They did not remove the lump and my condition has worsened” – 0006*

### Financial burden

Some participants mentioned their concerns regarding the cost of treatment. One woman did not return to KATH for further treatment after the first chemotherapy cycle and the other was worried about accumulating debt from her treatment.

*“Due to financial problems, I did not come back to KATH, I went to a peripheral hospital instead” – 0003*

*“I am just worried about the debt I have to pay, I was building but it is halted because all the money has been used” - 0008*

## Discussion

This study is unique in employing a longitudinal design to explore the expectations of metastatic breast cancer patients from their treatment. The benefit of chemotherapy for advanced cancer treatment is not guaranteed for every patient. The unwanted side effects of cytotoxics raise concerns about the effectiveness of therapies that slow tumour progression or prolong survival, but that result in a significant reduction in quality of life and overall well-being. Despite the lack of evidence to support the practice, chemotherapy is used widely in cancer patients with poor performance status and disease progression.^12^,^13^,^23^ While multiple lines of treatment may be appropriate for some patients and desired by others, realistic expectations for potential treatment benefits must be kept in mind during discussions with patients and their families.

Most participants in this study indicated healing as their treatment goal. Some expected the treatment to avert death. Patients with metastatic breast cancer have been known to associate death with their diagnosis, with fear that the disease will worsen and they will die from it.^15^ Treatment for metastatic cancer is, however, mainly palliation rather than cure. The expectation of healing or cure among participants will be considered medically unfeasible. Looking for a cure as the outcome for treating metastatic cancer has been expressed by cancer patients in other studies^24^–^27^ including Ghana.^28^,^29^ Reasons for this may include inadequate communication to disclose the diagnosis and prognosis, inadequate communication even when it was present, patients' inability to remember the discussion, or physicians being overly optimistic.^15^,^30^,^31^ Inadequate communication could be due to lack of indigenous terms to communicate in lay language, and poor health literacy among the population as seen in Kenya.^32^ Challenges with clinical consultations may also lead to patients forming unfeasible treatment expectations. In Kenya, health professionals reported issues such as lack of privacy to communicate, specialists not being available and so deferring communication to persons who may not have the right information to share, and short encounters that leave patients no time to process and accept the information given.^32^ The patient burden in the KATH breast and oncology clinics makes short consultation periods inevitable and, as a result, it is not unexpected that information will be inadequate. This is similar in other public facilities treating breast cancer. All clinical staff that patients encounter during their treatment journey must have training in undertaking difficult conversations related to prognosis to guide patients realistically along their treatment pathways. Patient counselling needs to be an ongoing process rather than an ‘event’. Involving family in the process may also be helpful.^32^ The goal is not to take away hope but to help patients make informed decisions and choices about their care pathways.

Spirituality and religion are reported to influence how patients cope with cancer diagnosis and find meaning during their cancer journey in African contexts, including Ghana, with patients believing in and anticipating supernatural healing.^33^ Religious beliefs about healing are not held only by patients, but also by health providers. Cancer patients in Nigeria reported health professionals presented prognostic information with a lot of optimism, including making some patients believe in God's miracle for a cure, or withheld prognostic information completely.^34^ Such situations feed into already existing cultural and religious beliefs of healing through prayer for cancer,^32^ and ultimately unfeasible expectations from treatment.

Symptom burden experienced by women with MBC includes hopelessness, anxiety, depression, fear, sleeplessness, fatigue, pain, breathlessness, swollen arms, mobility problems, and loss of appetite.^28^,^35^–^37^ Participants in this study expected improvement in their symptoms, such as reduction in arm and breast swelling, pain relief, and not experiencing fatigue. While some experienced improvement, others saw no change, and some had deteriorated. Similar trends of improvement, no change, and deterioration over time have been reported for breast cancer patients, including those with MBC undergoing treatment in Kenya and Senegal.^38^,^39^ One participant wanted her breast “cut off” to reduce her pain. This is rather contradictory to findings of fear of mastectomy influencing treatment decision-making of patients with breast cancer in Ghana.^40^ Within the context of MBC, this may be expected for some women due to the desperation at this point to get well. A prior study in KATH about the health-seeking behaviour of women with MBC found that at the ‘tipping point’ of seeking care, women are willing to do anything to return to their previous state of good health and function.^29^ In this study, this finding was confirmed again, where participants expected the treatment to allow them to return to functions and roles such as looking after their family, continuing their business, and even attending social gatherings.

Among the 28 who were alive at follow-up, some admitted their symptoms had improved, but none had complete symptom resolution. Some MBC patients do not expect complete resolution of their symptoms with treatment^34^, and some have discontinued treatment due to the side effects and lack of confidence in the treatment they were receiving.^15^ Women must be supported when deciding to stop cancer treatment because chemotherapy use has been associated with worse Quality of Death (QoD) among patients with good performance status, and no improvement in those with poor performance status.^10^ Complete resolution with problems like arm or breast swelling and lumps may be impossible, however every effort to control distressing symptoms like pain and breathlessness must be pursued with appropriate pharmacological and non-pharmacological interventions available. Prioritising the treatment of symptoms that are most bothersome has been found to have the greatest functional impact in women with MBC.^41^ Such goals can be achieved through effective conversations about diagnosis, prognosis and realistic treatment goals.

That half of the participants had died at the 3 months follow up suggests the expectations of cure and avoiding death had not been met for them. This high mortality rate suggests patients with MBC present to the hospital late and in poor health states. Although reporting outcomes over longer periods, the poor survival rates in this study reflect similar findings in other African countries, such as 1-year survival of 21.7% in Nigeria^42^, 3-year survival of 31.3% in Kenya^43^, and 5-year survival of 37.9% in Uganda.^44^ In recent times, overall survival for de novo MBC has improved due to the availability of novel treatment modalities.^45^ Catching up with prevailing global statistics in Ghana and similar settings will require addressing challenges like cost, access and health-seeking behaviour. 46,47

Financial costs resulting from out-of-pocket payments are a major stressor at the end of life and are associated with worse QoL.^42^–^45^ Significant costs for both patients and society may be due to supportive medications, hospitalisations to manage acute treatment-related toxicities, and transport costs.^9^ In this study, the cost of treatment led one participant to discontinue treatment. Cost of treatment remains a significant barrier to treatment initiation and compliance among breast cancer patients in Ghana and other African settings like Nigeria and Uganda.^28^,^40^,^46^ Indeed, cancer treatment costs can be up to five times the yearly income of an African worker.^47^ There may not be immediate solutions to treatment cost-related issues. But even in poor resource settings where health budgets are low, the ability to provide universal health coverage for cancer care is possible, as has been demonstrated in Namibia, allowing nearly all breast cancer patients to receive the care they need.^46^ Health systems and, by extension, governments need to commit more to healthcare than they already do.

### Limitations and Recommendations

The scope of the data in this study was limited and did not include the rationale for choosing systemic or supportive cancer treatment, which was determined solely by clinicians and the patient. Also, transcripts were not segregated by the type of treatment patients received or by sociodemographic characteristics for the analysis. We recommended that further studies explore how these themes shape patient expectations, offering more granular insights to guide clinicians in conducting effective patient conversations. The high death rate of 50% in this study is a source of bias. Considering the underlying disease of MBC, this is not unexpected, as poor health can contribute to high dropout in longitudinal studies,^48^ In this case, due to death. Death in patients with underlying severe illness, such as MBC, may be unpreventable, and interventions to mitigate such bias arising from such inevitable occurrence in future studies could include multi-centre data collection and a longer timespan of participant recruitment. The high death rate notwithstanding, 28 interviews at the follow-up period were adequate to achieve thematic saturation.^49^

## Data Availability

The datasets used and/or analysed during the current study are available from the corresponding author on reasonable request.

## Conclusion

The findings highlight the need to improve conversations held with patients with MBC to guide their expectations from the treatment they receive, whether cancer specific or supportive treatment only. This is very important within the context where the study was conducted, where many patients present late, prognosis is poor (50% death rate at 3 months in this study population), and cost remains a challenge with accessing and continuing treatment. The need for realistic expectations to guide pragmatic treatment decisions cannot be understated.
